# Field Evaluation of Vinegar-Based Attractants and Their Volatile Profiles for Monitoring *Drosophila suzukii* (Diptera: Drosophilidae) in Blackberry and Blueberry Orchards

**DOI:** 10.3390/plants15152264

**Published:** 2026-07-24

**Authors:** Adnan Tusun, Gülsevim Tiring

**Affiliations:** 1 Department of Plant Protection, Faculty of Agriculture, Çukurova University, Adana 01330, Türkiye; 2 Department of Plant and Animal Production, Vocational School of Kozan, Çukurova University, Adana 01500, Türkiye; gtiring@cu.edu.tr

**Keywords:** Spotted-wing drosophila (*Drosophila suzukii*), fermentation bait, GC-MS, integrated pest management (IPM)

## Abstract

The spotted-wing drosophila, *Drosophila suzukii* (Matsumura, 1931) (Diptera: Drosophilidae), is an invasive pest causing substantial economic losses in berry production worldwide. Reliable monitoring is essential for effective integrated pest management; however, information on the field performance of locally produced vinegar-based attractants remains limited. This study compared the attractiveness of four vinegar-based attractants (homemade grape vinegar, homemade hawthorn vinegar, homemade apple vinegar, and commercial apple vinegar) for monitoring *D. suzukii* in blackberry (*Rubus* spp.) and blueberry (*Vaccinium* spp.) orchards in southeastern Türkiye during the 2023 and 2024 growing seasons. In addition, the volatile organic compound (VOC) profiles of the attractants were characterized using headspace solid-phase microextraction coupled with gas chromatography–mass spectrometry (HS-SPME/GC–MS). A total of 80,571 adult flies were captured during the study. Significant differences among attractants were detected in all host plant–year combinations (one-way ANOVA, *p* < 0.001). Generalized linear mixed model analysis further demonstrated significant effects of the host plant, attractant type, sampling week, and year on weekly trap captures (*p* < 0.05). Homemade grape vinegar consistently produced the highest trap captures, whereas commercial apple vinegar showed the lowest trapping performance. Fourteen volatile compounds belonging mainly to carboxylic acids, alcohols, esters, aldehydes, and monoterpenes were identified, revealing differences in the volatile profiles of the evaluated attractants. Overall, homemade grape vinegar proved to be the most effective vinegar-based attractant under the conditions of this study and represents a practical, low-cost option for monitoring *D. suzukii* in berry orchards. Further studies incorporating replicated chemical analyses and behavioural bioassays are needed to clarify the contribution of individual volatile compounds to insect attraction.

## 1. Introduction

Invasive insect pests pose an increasing threat to agricultural ecosystems worldwide, leading to substantial economic losses and reduced crop productivity [1]. Among these pests, the spotted-wing drosophila, *Drosophila suzukii* (Matsumura, 1931) (Diptera: Drosophilidae), has emerged as one of the most destructive pests affecting berry, stone fruit, and viticulture production systems worldwide. The species was first recorded on cherry in Yamanashi, Japan, in 1916 [2] and was later detected in Türkiye on strawberry in Erzurum in 2014 [3,4].

Unlike most other drosophilid species, which primarily develop on overripe or decaying fruit, female *D. suzukii* are capable of laying eggs in intact, ripening fruits due to their specialized serrated ovipositor [5,6]. This unique biological adaptation allows the insect to exploit a wide range of host plants, including species within *Prunus* (cherry, peach, plum), *Rubus* (raspberry, blackberry), *Fragaria* (strawberry), *Vaccinium* (blueberry), and *Vitis* (grape), as well as other soft-skinned fruit crops [7]. Larval feeding within fruit tissues causes internal damage, accelerates decomposition, and increases susceptibility to secondary microbial infections, ultimately rendering fruits unmarketable [6]. Under favorable environmental conditions, infestations can result in yield losses reaching up to 80% [8,9].

Chemical control remains the primary management strategy against *D. suzukii*; however, the pest’s short generation time and high reproductive capacity often necessitate frequent insecticide applications, raising concerns related to cost, resistance development, and environmental impact [10,11,12]. Consequently, integrated pest management (IPM) approaches incorporating cultural and monitoring-based strategies are increasingly emphasized. Cultural practices such as field sanitation, including the removal of infested and overripe fruits as well as alternative host plants, can help reduce population buildup [6,9,13,14]. Among IPM components, effective monitoring is essential for early detection, population tracking, and timely implementation of control measures. Reliable monitoring also contributes to sustainable agriculture by supporting timely pest management decisions and reducing unnecessary insecticide applications.

Monitoring traps are widely used to assess the spatial distribution and seasonal dynamics of *D. suzukii* populations and to evaluate the effectiveness of management strategies. Since its invasion in Europe and the Americas, numerous trap designs and attractant formulations have been tested for monitoring purposes [15,16]. Fermentation-based attractants have received particular attention, including apple cider vinegar [15,17,18], brown rice vinegar [19,20], wine [21], and yeast-based baits [18,22,23]. Additionally, combinations of yeast, wine, and vinegar [21,24,25], synthetic volatile blends derived from fermentation [26,27,28], plant-based attractants combined with fruit juices [9], and commercial lures [29,30,31] have also been evaluated.

Despite the extensive research conducted on *D. suzukii* attractants, relatively little information is available regarding the attractiveness of locally produced fruit vinegars and the volatile compounds associated with their attractiveness. Furthermore, studies integrating field evaluations of vinegar-based attractants with detailed analyses of their volatile organic compound (VOC) profiles remain scarce. Improved understanding of the volatile composition of these attractants may facilitate the development of cost-effective, locally available, and efficient monitoring tools for *D. suzukii* management.

In this context, the present study aimed to (i) evaluate the trapping performance of four vinegar-based attractants (grape vinegar, apple vinegar, hawthorn vinegar, and commercial apple vinegar), (ii) compare population densities of *D. suzukii* between blackberry (*Rubus* spp.) and blueberry (*Vaccinium* spp.) orchards, and (iii) characterize the VOC profiles of the tested attractants to provide descriptive information on potential differences associated with trapping performance. The findings provide practical guidance for selecting inexpensive, locally available attractants for field monitoring of *D. suzukii* while establishing a foundation for future studies investigating the role of volatile compounds in insect attraction.

## 2. Results

### 2.1. Seasonal Captures of Drosophila suzukii

A total of 80,571 adult *D. suzukii* were captured during the study period across both berry production systems (Table 1). Of the total captures, 46,529 adults (57.8%) were collected from blueberry orchards, whereas 34,042 adults (42.2%) were collected from blackberry orchards.

Across both host plants, trap catches were higher in 2023 than in 2024. In blueberry orchards, 24,695 adults (53.1%) were recorded in 2023 and 21,834 (46.9%) in 2024, whereas blackberry orchards recorded 17,599 adults (51.7%) and 16,443 adults (48.3%) during the respective years.

### 2.2. Seasonal Cumulative Captures According to Attractant Type

The mean seasonal cumulative captures of *D. suzukii* adults differed significantly among attractant treatments in both host plants and years (Table 1). In all experimental combinations, homemade grape vinegar consistently produced the highest cumulative captures, whereas commercial apple vinegar resulted in the lowest captures.

In blackberry orchards, grape vinegar captured 1360.8 ± 30.66 and 1279.8 ± 17.51 adults trap^−1^ during 2023 and 2024, respectively. In blueberry orchards, grape vinegar also produced the highest cumulative captures (1923.0 ± 53.49 and 1705.0 ± 20.58 adults trap^−1^ in 2023 and 2024, respectively).

Within each host plant and year, one-way ANOVA showed significant differences among the four vinegar-based attractants (blackberry 2023: F = 71.353, df = 3, 15, *p* < 0.001; blackberry 2024: F = 67.290, df = 3, 15, *p* < 0.001; blueberry 2023: F = 52.270, df = 3, 15, *p* < 0.001; blueberry 2024: F = 73.237, df = 3, 15, *p* < 0.001). Post hoc comparisons using Tukey’s HSD test indicated that homemade grape vinegar produced significantly higher cumulative trap captures than the other attractants in all host plant–year combinations (Table 1).

A three-way ANOVA was performed to evaluate the effects of host plant, attractant type, and year on seasonal cumulative trap catches. Host plant significantly affected cumulative adult captures (F_1_,_2224_ = 56.27, *p* < 0.001), indicating differences in *D. suzukii* abundance between blackberry and blueberry orchards. Attractant type also significantly influenced cumulative trap captures (F_3_,_2224_ = 20.79, *p* < 0.001), whereas year had a smaller but still significant effect (F_1_,_2224_ = 5.82, *p* = 0.016). No significant interactions were detected between host plant and attractant (F_3_,_2224_ = 0.60, *p* = 0.614), host plant and year (F_1_,_2224_ = 1.05, *p* = 0.306), attractant and year (F_3_,_2224_ = 0.05, *p* = 0.986), or among all three factors (F_3_,_2224_ = 0.04, *p* = 0.989). These results indicate that host plant, attractant type, and year each had significant independent effects on seasonal cumulative adult captures, whereas none of their interactions significantly influenced trap catches.

### 2.3. Generalized Linear Mixed Model Analysis

To account for repeated weekly observations and the overdispersed nature of trap-count data, weekly captures were analysed using a generalized linear mixed model (GLMM) with a negative binomial distribution and log link function (Table 2).

The GLMM revealed significant effects of host plant (Wald χ^2^ = 54.43, df = 1, *p* < 0.001), year (Wald χ^2^ = 5.38, df = 1, *p* = 0.020), attractant type (Wald χ^2^ = 20.82, df = 3, *p* < 0.001), sampling week (Wald χ^2^ = 35,532.31, df = 34, *p* < 0.001), and the attractant × sampling week interaction (Wald χ^2^ = 54.43, df = 102, *p* < 0.001) on weekly adult captures (Table 2).

After accounting for year, attractant type, and sampling week, weekly captures were significantly higher in blueberry than in blackberry orchards (IRR = 1.36, 95% CI = 1.25–1.47, *p* < 0.001), corresponding to an approximately 36% higher expected capture rate. A significant year effect was also detected, with weekly captures in 2024 being approximately 9% lower than those in 2023 (IRR = 0.91, 95% CI = 0.84–0.99, *p* = 0.020).

Sampling week had the strongest effect on adult abundance. Compared with week 1, captures did not differ significantly during weeks 2–14 (*p* > 0.05), but increased significantly from week 15 onward. The highest expected capture rates occurred during weeks 28–35, with week 35 showing an approximately 12.9-fold higher capture rate than week 1 (IRR = 12.92).

Estimated marginal means indicated that homemade grape vinegar consistently produced the highest weekly captures, whereas commercial apple vinegar produced the lowest. Pairwise comparisons showed that homemade grape vinegar captured significantly more adults than homemade apple vinegar and commercial apple vinegar (*p* < 0.05), whereas no significant difference was detected between homemade grape vinegar and homemade hawthorn vinegar.

The significant attractant × sampling week interaction indicated that differences among attractants varied throughout the sampling period. Although attractants performed similarly during the early weeks, the superiority of homemade grape vinegar became increasingly evident as *D. suzukii* populations increased later in the season.

### 2.4. Seasonal Population Dynamics

**Figure 1:**
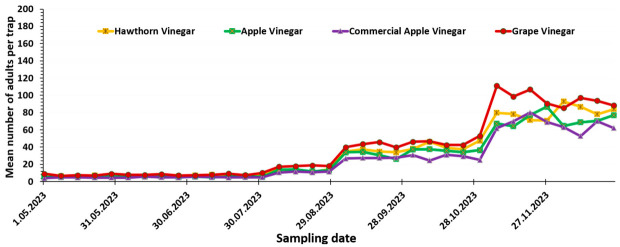
Seasonal population dynamics of *Drosophila suzukii* in a blackberry orchard during the 2023 growing season as monitored using traps baited with four vinegar-based attractants (hawthorn vinegar, apple vinegar, commercial apple vinegar, and grape vinegar). Values represent the weekly mean number of adults captured per trap based on four trap replicates (*n* = 4).

**Figure 2:**
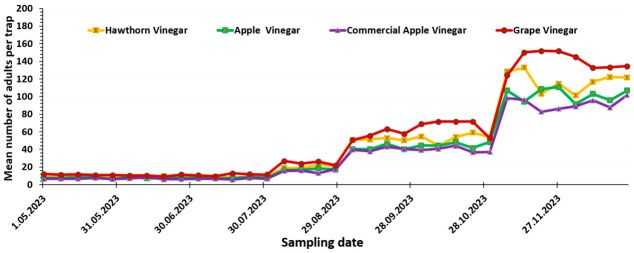
Seasonal population dynamics of *Drosophila suzukii* in a blueberry orchard during the 2023 growing season as monitored using traps baited with four vinegar-based attractants (hawthorn vinegar, apple vinegar, commercial apple vinegar, and grape vinegar). Values represent the weekly mean number of adults captured per trap based on four trap replicates (*n* = 4).

**Figure 3:**
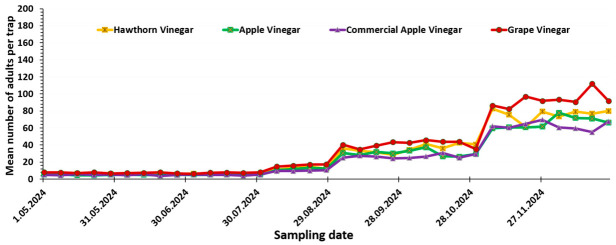
Seasonal population dynamics of *Drosophila suzukii* in a blueberry orchard during the 2024 growing season as monitored using traps baited with four vinegar-based attractants (hawthorn vinegar, apple vinegar, commercial apple vinegar, and grape vinegar). Values represent the weekly mean number of adults captured per trap based on four trap replicates (*n* = 4).

**Figure 4:**
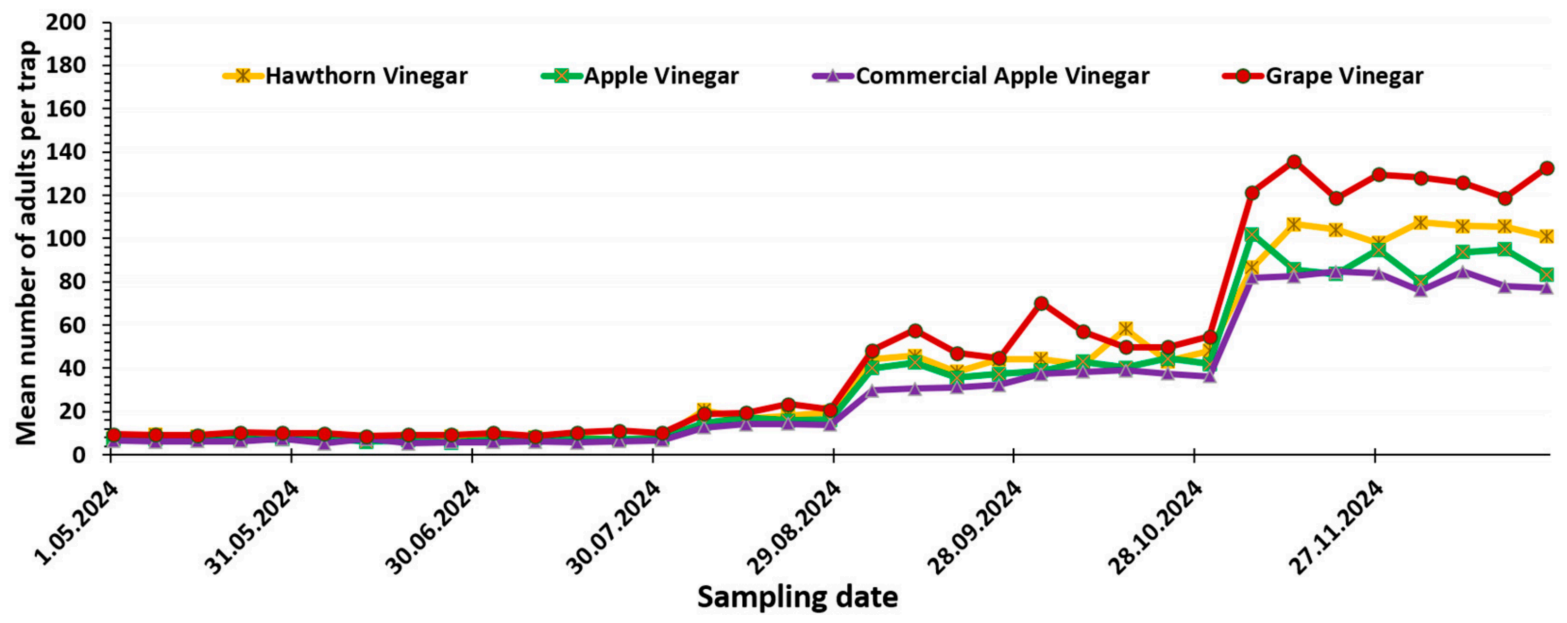
Seasonal population dynamics of *Drosophila suzukii* in a blackberry orchard during the 2024 growing season as monitored using traps baited with four vinegar-based attractants (hawthorn vinegar, apple vinegar, commercial apple vinegar, and grape vinegar). Values represent the weekly mean number of adults captured per trap based on four trap replicates (*n* = 4).

The seasonal population dynamics of *D. suzukii* in blackberry and blueberry orchards are presented in Figure 1, Figure 2, Figure 3 and Figure 4. Population trends were generally similar between the two study years, although overall adult captures were slightly lower in 2024 than in 2023. In both blackberry and blueberry orchards, adult captures remained low during the early part of the growing season, followed by a gradual increase from August onwards. Trap catches increased markedly during September and October and reached their highest levels during November and December before declining at the end of the sampling period. Across both years, greater numbers of *D. suzukii* adults were captured in blueberry orchards than in blackberry orchards. Although all attractants followed comparable seasonal trends, homemade grape vinegar generally yielded the highest weekly captures throughout the sampling period, whereas commercial apple vinegar consistently yielded the lowest captures across both host plants and years.

### 2.5. Volatile Compounds of Vinegar Attractants

**Figure 5:**
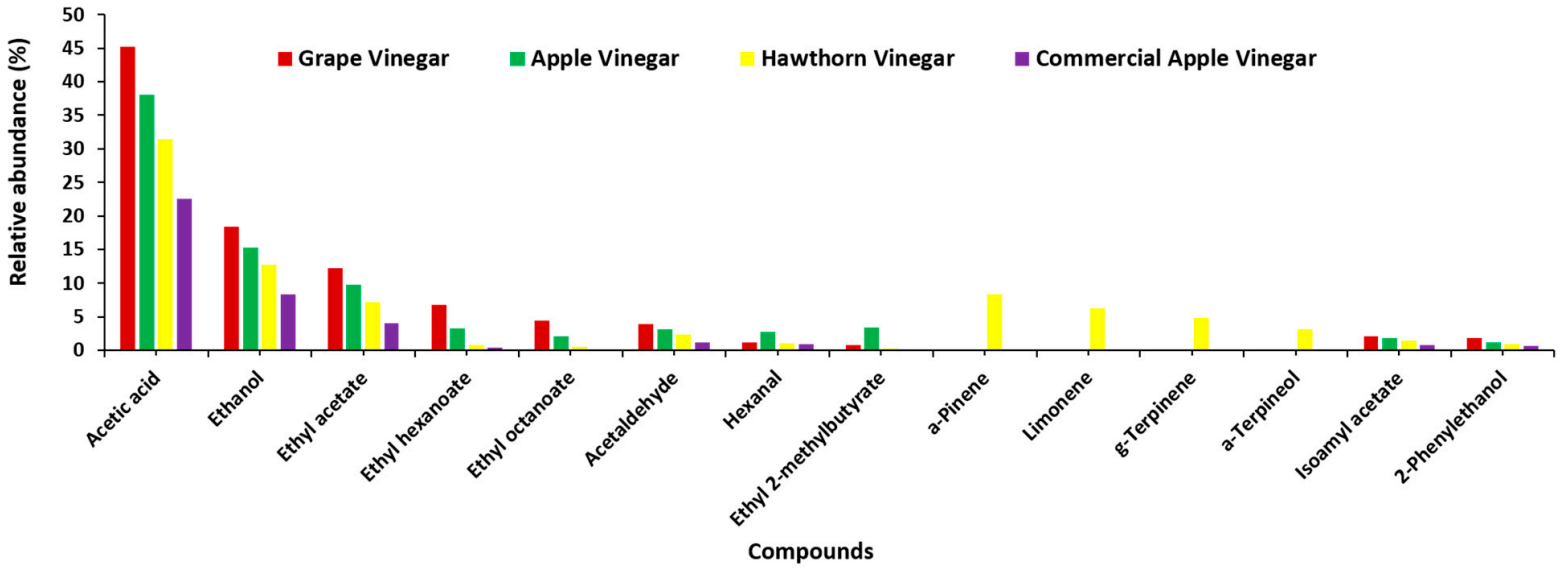
Relative abundance (%) of volatile organic compounds (VOCs) identified by HS-SPME/GC–MS in homemade hawthorn vinegar, homemade apple vinegar, commercial apple vinegar, and homemade grape vinegar. Relative abundances were calculated from normalized GC–MS peak areas. The VOC profiles are descriptive and are based on one representative sample from each attractant; therefore, no statistical comparisons were performed.

A total of 14 volatile organic compounds (VOCs) were identified across the four attractant types, belonging mainly to carboxylic acids, alcohols, esters, aldehydes, and monoterpenes. The overall proportion of identified compounds varied substantially among formulations, ranging from 39.3% in commercial apple vinegar to 97.0% in grape vinegar (Figure 5).

Acetic acid was the dominant compound in all vinegar types, with the highest relative abundance observed in grape vinegar (45.2%), followed by apple vinegar (38.1%), hawthorn vinegar (31.4%), and commercial apple vinegar (22.6%). Ethanol was the second most abundant compound across all samples, showing a similar decreasing trend from grape vinegar (18.4%) to commercial apple vinegar (8.3%) (Figure 5).

Esters represented the major group of fermentation-related volatiles. Ethyl acetate was present in all formulations and showed the highest level in grape vinegar (12.3%). Other esters, including ethyl hexanoate, ethyl octanoate, ethyl 2-methylbutyrate, isoamyl acetate, and ethyl octanoate derivatives, were generally more abundant in grape vinegar compared to the other attractants (Figure 5).

Aldehydes such as acetaldehyde and hexanal were detected at lower levels across all samples. Monoterpene compounds (α-pinene, limonene, γ-terpinene, and α-terpineol) were detected only in hawthorn vinegar, indicating a distinct plant-derived volatile signature for this formulation (Figure 5).

Overall, grape vinegar exhibited the highest diversity and relative abundance of fermentation-related volatiles, while commercial apple vinegar showed the lowest overall VOC complexity (Figure 5).

## 3. Discussion

Since its invasion of new fruit-producing regions, *D. suzukii* has become an abundant and economically important pest in many parts of the world, including North America and Europe [6,32]. Consequently, considerable research has focused on developing effective monitoring programmes, including the evaluation and standardization of trapping systems and attractants [15]. The development of a widely accepted monitoring tool requires not only high trapping efficiency but also consideration of material costs, ease of handling, durability, and applicability across different crops and production systems [16]. In this context, the present study evaluated the effectiveness of four vinegar-based attractants and examined their VOC profiles in blackberry and blueberry orchards in Türkiye.

The seasonal population dynamics observed in the present study were generally consistent with previous reports from Türkiye. *Drosophila suzukii* captures remained relatively low from May to August and increased markedly during the subsequent months. Similarly, Başpınar et al. (2022) reported low drosophilid abundance during the summer period and suggested that high temperatures, low relative humidity, and limited precipitation may negatively affect drosophilid populations [33]. Likewise, Kasap and Özdamar (2019) observed a substantial increase in *D. suzukii* populations after September, with peak captures occurring during November and December [34]. In addition to climatic factors, Zengin and Karaca (2019) suggested that the availability of natural food resources in the environment may reduce the attractiveness of vinegar-baited traps, as *D. suzukii* adults may preferentially exploit naturally available hosts [35]. Therefore, the low captures recorded during the summer months in the present study may have resulted from a combination of unfavorable climatic conditions and the availability of alternative food and oviposition resources within the orchards. The subsequent increase in captures observed from late summer onwards likely reflects the occurrence of more favorable environmental conditions for population development and adult activity, consistent with the seasonal trends reported in previous studies.

Across both crops and study years, homemade grape vinegar produced the highest cumulative trap captures, significantly exceeding those obtained with hawthorn vinegar, homemade apple vinegar, and commercial apple vinegar. Similar studies have demonstrated that fermentation-based attractants are among the most effective lures for *D. suzukii* because adults rely heavily on microbial fermentation cues when locating food sources and suitable habitats [21,26]. The superior performance of grape vinegar observed in the present study may be associated with differences in its volatile composition, which may more closely resemble the odor profile of naturally fermenting substrates exploited by *D. suzukii*.

The factorial ANOVA revealed that host plant, attractant type, and year all significantly influenced the number of *D. suzukii* adults captured. Among these factors, host plant and attractant type exhibited strong and consistent effects, indicating that trap captures were primarily driven by crop system and bait formulation. Although year also had a statistically significant effect, its influence on trap captures was less pronounced than that of host plant and attractant type.

Importantly, no significant host plant × attractant interaction was detected, demonstrating that the relative ranking of attractants remained stable across both blueberry and blackberry orchards. Similarly, the lack of significant interactions involving year indicates that the effects of attractants and host plants were consistent over time. Overall, these results suggest that attractant performance is robust across different host plants and sampling years, supporting their reliability for monitoring *D. suzukii* populations under variable field conditions.

The VOC analyses provided descriptive information that may help explain the observed differences in trapping performance. Acetic acid was the dominant volatile compound in all vinegar formulations, followed by ethanol. Notably, the analysed grape vinegar sample showed the highest relative abundance of both compounds, and grape vinegar also produced the highest trap captures under field conditions. Acetic acid and ethanol are widely recognized as core fermentation-related attractants for *D. suzukii*. Landolt et al. (2012a) demonstrated that combinations of acetic acid and ethanol attract significantly more adults than either compound alone, indicating a synergistic interaction between these fermentation products [21]. Similarly, Cha et al. (2012) identified these compounds as major constituents of attractive fermentation-derived odor blends [26]. The relatively higher abundance of acetic acid and ethanol observed in the analysed grape vinegar sample may have contributed to its higher trapping performance, although no causal relationship was tested in the present study.

However, the superiority of grape vinegar cannot be explained solely by its higher concentrations of acetic acid and ethanol. Previous studies have shown that *D. suzukii* responds not only to individual compounds but also to complex mixtures of fermentation-derived volatiles. Cha et al. (2012) [26] reported that several individual esters, including ethyl acetate and isoamyl acetate, reduced attraction when added separately to acetic acid–ethanol mixtures. In contrast, synthetic blends containing multiple fermentation-associated compounds, such as acetoin and 2-phenylethanol, significantly increased attractiveness [26]. These findings indicate that attraction is determined primarily by the composition and balance of volatile blends rather than by the presence of any single compound.

These observations are consistent with the VOC profiles obtained in the present study. In addition to exhibiting the highest relative abundances of acetic acid and ethanol, grape vinegar showed the greatest abundance and diversity of ester compounds. Such complex fermentation-derived odor bouquets may more accurately mimic naturally fermenting fruits and microbial substrates that serve as feeding resources for adult flies. Consequently, the higher captures obtained with grape vinegar may reflect differences in the overall volatile blend rather than the effect of any individual compound.

Commercial apple vinegar exhibited the lowest trapping performance throughout the study and also showed the lowest overall VOC complexity. In particular, the analysed commercial apple vinegar sample showed lower relative abundances of acetic acid, ethanol, and ester compounds than the analysed grape vinegar sample. Previous studies have suggested that the attractiveness of fermentation-based baits increases with the complexity and intensity of fermentation-associated odor blends [26,27]. The reduced effectiveness of commercial apple vinegar observed in the present study is therefore consistent with the relatively less complex VOC profile observed in the analysed sample.

Although homemade grape vinegar exhibited the highest trapping efficiency in the present study, vinegar-based fermentation baits are generally considered non-specific attractants and may capture a wide range of non-target insects in addition to *D. suzukii*.

Although homemade grape vinegar showed the highest trapping performance under the conditions of the present study, monitoring programmes should also consider the potential capture of non-target insects. Previous studies have shown that fermentation-based baits may attract numerous non-target Diptera and other insects, whereas chemically defined lures can substantially reduce by-catch while maintaining monitoring efficiency [36]. Likewise, studies on other insect pests have demonstrated that trap characteristics, including colour, trap height, placement, and structural design, can influence monitoring efficiency and trap selectivity by affecting captures of both target and non-target arthropods [37,38]. Therefore, further optimization of trap design may contribute to improving monitoring selectivity while maintaining effective detection of *D. suzukii*. In addition, deploying traps only during periods when fruits are susceptible to infestation, removing them after harvest, and reducing the diameter of trap entry holes may further minimize captures of larger non-target insects.

Overall, homemade grape vinegar represents an inexpensive and readily available attractant for monitoring *D. suzukii* in berry orchards. Nevertheless, because fermentation-based baits may also attract non-target insects, future research should focus on improving lure selectivity while maintaining trapping efficiency. Such studies will contribute to the development of more sustainable monitoring tools for integrated pest management programs. The present findings provide a practical basis for incorporating locally produced vinegar-based attractants into regional monitoring programs for *D. suzukii*.

## 4. Materials and Methods

### 4.1. Study Location

**Figure 6:**
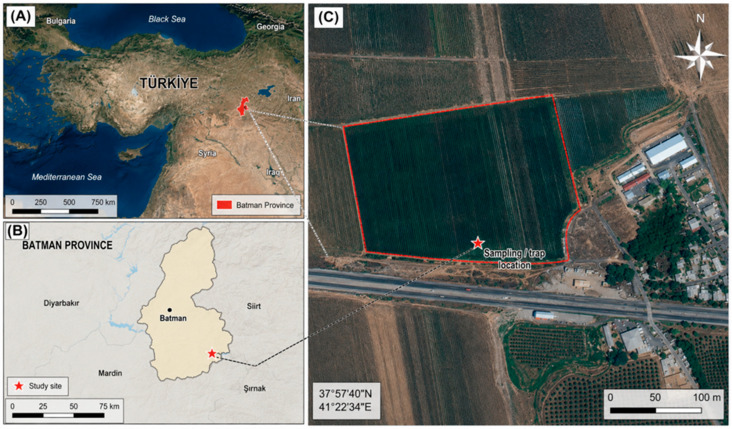
Location of the study area. (**A**) Geographic location of Batman Province in southeastern Türkiye. (**B**) Location of the experimental orchards within Batman Province (red star). (**C**) Satellite image of the blackberry and blueberry orchards showing the study site and trap location (37°57′40″ N, 41°22′34″ E), where *Drosophila suzukii* populations were monitored during the 2023–2024 growing seasons.

The study was conducted during 2023 and 2024 in blackberry and blueberry orchards located in Gökdoğan Village, Kurtalan District, Siirt Province, southeastern Türkiye (Figure 6). The orchards, each covering approximately 50 decares, are situated along the banks of the Yanarsu Stream at 37°57′40″ N and 41°22′34″ E.

The study area is characterized by a semi-arid continental climate, with hot and dry summers and cool, rainy winters (Table 3). The orchards are established in a valley ecosystem influenced by the Yanarsu Stream, which provides favorable humidity conditions and irrigation resources for fruit production.

### 4.2. Trapping and Population Monitoring

The population dynamics of *D. suzukii* were monitored in blackberry and blueberry orchards during the 2023 and 2024 growing seasons. Four vinegar-based attractants were evaluated: homemade grape vinegar, homemade hawthorn vinegar, homemade apple vinegar, and commercial apple vinegar. Each attractant was replicated four times in each orchard, resulting in 16 traps per orchard (4 attractants × 4 replicates) and a total of 32 traps.

Homemade grape, apple, and hawthorn vinegars were obtained from local household producers in southeastern Türkiye. These vinegars had been prepared using traditional natural fermentation methods commonly practiced in Türkiye. To minimize variation among sampling dates, each homemade vinegar type was obtained from a single production batch and used throughout the study. Commercial apple vinegar was purchased from a local supermarket.

**Figure 7:**
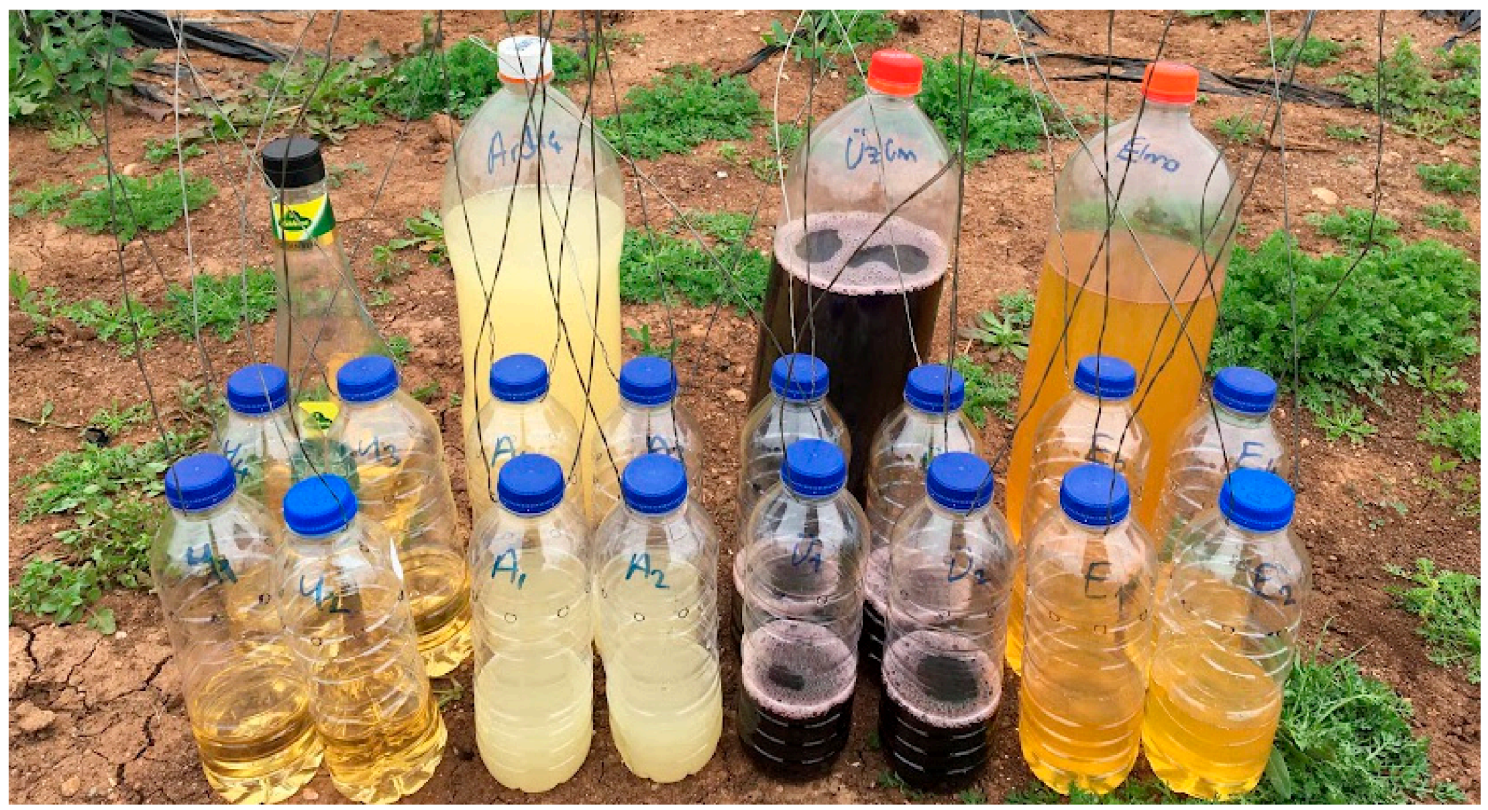
Vinegar-based attractants and trap design used for monitoring *Drosophila suzukii*. Transparent 0.5-L PET bottle traps containing 180 mL of attractant were baited with homemade grape vinegar, homemade hawthorn vinegar, homemade apple vinegar, or commercial apple vinegar and deployed in blackberry and blueberry orchards during the 2023 and 2024 growing seasons. The handwritten labels on the bottles indicate the attractant type: A = commercial apple vinegar, Üzüm = homemade grape vinegar, Alıç = homemade hawthorn vinegar, and Elma = homemade apple vinegar.

Traps consisted of transparent 0.5-L polyethylene terephthalate (PET) bottles (Figure 2), each containing 180 mL of the respective attractant (Figure 7). Four circular entrance holes (5 mm diameter) were made around the upper part of each bottle to allow adult flies to enter. Traps were suspended approximately 1.5 m above ground within the fruiting canopy.

Trap positions were randomly assigned at the beginning of the experiment. Traps were placed approximately 20 m apart and at least 10 m from the orchard edges to minimize interference among attractants and reduce edge effects. All traps were maintained under comparable canopy conditions with respect to canopy density and shading. The geographic position of each trap was recorded using a handheld GPS receiver.

Traps were installed in May 2023 at the onset of fruit ripening and inspected weekly throughout the study period. Captured insects were collected during weekly inspections and transported to the laboratory for identification and counting. The number of adults captured in each trap was recorded separately. Attractant solutions were replaced weekly during spring, summer, and autumn and every two weeks during winter to maintain trapping efficiency.

### 4.3. Analysis of Volatile Profiles of Fermented Liquid Attractants

The volatile organic compound (VOC) profiles of the attractants used in the trapping experiments were characterized using headspace solid-phase microextraction coupled with gas chromatography–mass spectrometry (HS-SPME-GC-MS) using an Agilent 8890 gas chromatograph coupled to an Agilent 5977B mass selective detector (Agilent Technologies, Santa Clara, CA, USA). Homemade grape vinegar, homemade apple vinegar, homemade hawthorn vinegar, and commercial apple vinegar were sampled immediately before trap deployment to determine their initial volatile composition. All attractants were obtained from the same batch used throughout the field experiments. Following collection, samples were transferred into sealed amber glass vials and stored at −20 °C until analysis.

For HS-SPME extraction, 5 mL of each attractant was placed in a 20 mL headspace vial and sealed with a PTFE-lined silicone septum. Samples were equilibrated at 40 °C for 20 min. Subsequently, a 50/30 μm DVB/CAR/PDMS fiber (Supelco, Bellefonte, PA, USA) was exposed to the headspace for 40 min at 40 °C. The fiber was then thermally desorbed in the GC injector port at 250 °C for 5 min under splitless conditions.

Chromatographic conditions were as follows. An HP-5MS capillary column (30 m × 0.25 mm i.d. × 0.25 μm film thickness) was used for chromatographic separation. Helium was used as the carrier gas at a constant flow rate of 1.0 mL min^−1^. The oven temperature program was set at 40 °C for 3 min, increased to 250 °C at a rate of 5 °C min^−1^, and held for 5 min. Mass spectra were acquired in electron ionization (EI) mode at 70 eV over a scan range of m/z 35–350.

Volatile compounds were tentatively identified by comparison of their mass spectra with those contained in the NIST 2020 Mass Spectral Library (National Institute of Standards and Technology, Gaithersburg, MD, USA). Only compounds with a spectral match quality of at least 80% were considered. Relative abundances were expressed as percentage peak areas normalized to the total ion chromatogram (TIC). Since no internal standard was used, the reported values represent semi-quantitative relative abundances rather than absolute concentrations. A single sample from each attractant type was analyzed to characterize the initial VOC profile of the attractants used in the field study. Therefore, VOC results were interpreted descriptively and were not subjected to statistical comparisons among attractant types.

### 4.4. Statistical Analysis

Statistical analyses were performed using R version 4.4.0 (R Core Team, Vienna, Austria) and IBM SPSS Statistics version 22.0 (IBM Corp., Armonk, NY, USA). Prior to analysis, data were tested for normality using the Shapiro–Wilk test and for homogeneity of variances using Levene’s test. Since the assumptions of parametric analysis were satisfied, differences among attractant treatments within each host plant and year were evaluated using one-way analysis of variance (ANOVA). When significant differences were detected, means were separated using Tukey’s honestly significant difference (HSD) test at *p* < 0.05.

To evaluate the combined effects of host plant, attractant type, and year on seasonal cumulative captures of *D. suzukii*, a three-way factorial analysis of variance (ANOVA) was performed. Host plant (two levels), attractant type (four levels), and year (two levels) were included as fixed factors. The main effects and all possible interaction terms (host plant × attractant type, host plant × year, attractant type × year, and host plant × attractant type × year) were tested. Statistical significance was declared at *p* < 0.05.

Because adult trap catches were recorded repeatedly from the same traps throughout the sampling season and the response variable consisted of overdispersed count data, weekly captures were additionally analysed using a generalized linear mixed model (GLMM) with a negative binomial distribution and log link function. Host plant, attractant type, year, and sampling week were included as fixed effects, while trap identity was included as a random effect to account for repeated measurements. Pairwise comparisons among attractants were performed using estimated marginal means with appropriate multiple-comparison adjustment. Statistical significance was accepted at *p* < 0.05.

## 5. Conclusions

This study demonstrated significant differences in the effectiveness of vinegar-based attractants for monitoring *D. suzukii* in blackberry and blueberry orchards over two consecutive growing seasons. Among the evaluated attractants, homemade grape vinegar consistently produced the highest trap captures, whereas commercial apple vinegar showed the lowest trapping efficiency. Three-way ANOVA and generalized linear mixed model analyses further confirmed that host plant, attractant type, year, and sampling week significantly influenced adult captures, while the absence of significant interaction effects indicated that the relative performance of the attractants remained consistent across host plants and years.

The descriptive VOC analysis showed differences in the volatile composition of the evaluated attractants, with the analysed grape vinegar sample exhibiting a greater diversity and relative abundance of fermentation-related compounds than the other attractants. Although these observations may help explain differences in trapping performance, no causal relationship between individual volatile compounds and insect attraction was established. Overall, homemade grape vinegar appears to be an inexpensive, readily available, and effective attractant for monitoring *D. suzukii* in berry orchards and may be suitable for incorporation into integrated pest management (IPM) programmes. Future studies should include replicated VOC analyses, behavioural bioassays, and assessments of non-target insect captures to improve lure selectivity and further clarify the role of volatile compounds in host attraction.

## Figures and Tables

**Table 1:** Mean (± SE) seasonal cumulative captures of *Drosophila suzukii* adults per trap baited with four vinegar-based attractants in blackberry and blueberry orchards during 2023 and 2024.

Attractant	Blackberry 2023	Blackberry 2024	Blueberry 2023	Blueberry 2024
Homemade hawthorn vinegar	1136 ± 21.75 b	1066.0 ± 25.83 b	1615.3 ± 38.27 c	1408.5 ± 32.10 d
Homemade apple vinegar	1013.8 ± 38.51 c	920.8 ± 19.48 c	1386.5 ± 35.36 c	1245 ± 40.81 c
Commercial apple vinegar	889.3 ± 21.90 d	844.5 ± 28.74 c	1250 ± 32.48 b	1099 ± 23.78 b
Homemade grape vinegar	1360.8 ± 30.66 a	1279.8 ± 17.51 a	1923 ± 53.49 a	1705 ± 20.58 a
F	71.353	67.290	52.270	73.237
sd	3, 15	3, 15	3, 15	3, 15
*p*	<0.001	<0.001	<0.001	<0.001

Values are presented as mean ± SE seasonal cumulative captures per trap (*n* = 4). Means followed by different lowercase letters within a column differ significantly according to Tukey’s HSD test (*p* < 0.05). One-way ANOVA was performed separately for each host plant and year.

**Table 2:** Results of the generalized linear mixed model (GLMM) for weekly *Drosophila suzukii* trap captures.

Fixed Effect	Wald χ^2^	df	*p*
Host plant	54.43	1	<0.001
Year	5.38	1	0.020
Attractant	20.82	3	<0.001
Week	35,532.31	34	<0.001
Attractant × Week	54.43	102	<0.001

**Table 3:** Long-term climatic characteristics of Batman Province, Türkiye (1952–2025).

Climatic Variable	Jan	Feb	Mar	Apr	May	Jun	Jul	Aug	Sep	Oct	Nov	Dec	Annual
Mean temperature (°C)	2.6	4.6	9.3	14.5	19.5	26.1	30.3	29.6	24.3	17.4	9.7	4.4	16.0
Mean maximum temperature (°C)	7.9	10.8	16.0	21.8	27.8	35.1	39.5	39.6	34.6	26.8	17.1	9.8	23.9
Mean minimum temperature (°C)	–1.4	0.0	3.6	7.9	11.4	15.9	20.2	19.7	15.1	10.1	4.2	0.6	8.9
Mean sunshine duration (h day^−1^)	3.4	4.8	5.6	7.5	9.2	11.7	12.0	11.3	9.9	7.1	5.2	3.1	7.6
Mean number of rainy days	10.74	9.97	11.46	11.01	8.30	2.12	0.42	0.35	1.06	5.58	7.35	9.93	78.3
Mean monthly precipitation (mm)	60.3	63.7	76.4	72.4	45.9	8.3	1.6	1.8	4.7	32.8	54.0	61.6	483.5
Highest recorded temperature (°C)	18.6	24.6	30.6	35.8	42.0	45.1	48.8	46.2	43.8	37.0	28.6	22.6	48.8
Lowest recorded temperature (°C)	–24.0	−22.2	–17.0	–9.0	0.9	5.0	11.8	11.1	4.4	–3.0	–7.8	–23.0	–24.0

Ref. [39] Turkish State Meteorological Service (MGM). Long-term climatic normals for Batman Province based on observations collected between 1952 and 2025. Long-term climatic data were included to characterize the environmental conditions of the study region.

## Data Availability

The original contributions presented in this study are included in the article. Further inquiries can be directed to the corresponding author.
